# Context-Dependent Encoding of Fear and Extinction Memories in a Large-Scale Network Model of the Basal Amygdala

**DOI:** 10.1371/journal.pcbi.1001104

**Published:** 2011-03-17

**Authors:** Ioannis Vlachos, Cyril Herry, Andreas Lüthi, Ad Aertsen, Arvind Kumar

**Affiliations:** 1 Bernstein Center for Computational Neuroscience Frieburg, Freiburg, Germany; 2 Neurocentre Magendie, Bordeaux Cedex, France; 3 INSERM U862, Bordeaux Cedex, France; 4 Friedrich Miescher Institute for Biomedical Research, Basel, Switzerland; 5 Department of Neurobiology and Biophysics, Faculty of Biology, University of Freiburg, Freiburg, Germany; John Radcliffe Hospital, United Kingdom

## Abstract

The basal nucleus of the amygdala (BA) is involved in the formation of context-dependent conditioned fear and extinction memories. To understand the underlying neural mechanisms we developed a large-scale neuron network model of the BA, composed of excitatory and inhibitory leaky-integrate-and-fire neurons. Excitatory BA neurons received conditioned stimulus (CS)-related input from the adjacent lateral nucleus (LA) and contextual input from the hippocampus or medial prefrontal cortex (mPFC). We implemented a plasticity mechanism according to which CS and contextual synapses were potentiated if CS and contextual inputs temporally coincided on the afferents of the excitatory neurons. Our simulations revealed a differential recruitment of two distinct subpopulations of BA neurons during conditioning and extinction, mimicking the activation of experimentally observed cell populations. We propose that these two subgroups encode contextual specificity of fear and extinction memories, respectively. Mutual competition between them, mediated by feedback inhibition and driven by contextual inputs, regulates the activity in the central amygdala (CEA) thereby controlling amygdala output and fear behavior. The model makes multiple testable predictions that may advance our understanding of fear and extinction memories.

## Author Summary

The amygdaloid complex is one of the key brain structures involved in fear-related processes. A typical way to study neural correlates of fear expression (e.g. freezing response) in the amygdala is to perform a fear conditioning paradigm, which yields a conditioned fear response. This response can be reversed by another procedure called fear extinction. Thanks to the experimental approaches to date we have some understanding about the putative roles of specific subnuclei within the amygdala in the formation of these fear and extinction memories. Here, we complement the experimental studies by providing a computational model that addresses the question of how fear and extinction memories are encoded in the amygdala, and specifically, in the basal nucleus (BA). We propose a specific neural mechanism to explain how the BA may integrate information about a salient, conditioned stimulus and the environment, thereby enabling it to switch the state of the animal from low to high fear and vice versa. We also provide possible explanations for various other behavioral findings, such as the recovery of fear after it had been extinguished (renewal). Finally, we make specific, experimentally testable predictions that need to be addressed in future work.

## Introduction

In classical fear conditioning an animal learns to associate an initially neutral stimulus (the conditioned stimulus, CS) with an aversive stimulus (the unconditioned stimulus, US) after paired exposure to the CS and the US. Subsequent repeated non-reinforced presentations of the CS alone result in a decline of the conditioned response, a process called fear extinction [1]. Fear extinction is a highly context-dependent process: the conditioned fear response returns when the animal is exposed to an extinguished CS outside the extinction context [2], [3].

Studies over the last decades have identified the amygdaloid complex as a key brain structure involved in both fear conditioning and extinction [4]–[6]. In the lateral nucleus of the amygdala (LA), signals carrying information about the CS and the US converge onto the same neurons where they become associated through activity-dependent plasticity mechanisms [7]–[9]. The LA can directly or indirectly influence activity in the central nucleus (CEA) [10], the major output nucleus of the amygdala that can trigger fear responses via its projections to the hypothalamus and to the brainstem [11]. The basal nucleus of the amygdala (BA) has been suggested to play an important role in contextual fear conditioning [12], [13], cued fear conditioning [14], fear extinction [15]–[17] and context-dependent fear renewal [17].

Recently, two distinct fear and extinction specific neuronal sub-populations in the BA have been identified [17]. The balance of activity between fear and extinction neurons was correlated with states of high and low fear, respectively. Moreover, pharmacological inactivation of the BA blocked the acquisition of fear extinction and context-dependent fear renewal, suggesting that BA fear and extinction neurons may underlie the induction of behavioral changes and contribute to the formation of fear and extinction memories.

These findings raise the question of what the potential mechanisms underlying the differential activation of these two neuronal sub-populations are. Here, we used a modeling approach based on *in vivo* physiological data to address this specific question and to draw more general conclusions on potential neural mechanisms involved in fear and extinction memories in the BA. In vivo stimulation of identified fear and extinction neurons revealed that the two neuronal populations receive differential functional input from the hippocampus and from the medial prefrontal cortex (mPFC) [17]. This finding could reflect anatomical specificity of inputs and/or selective functional plasticity of non-specific inputs. Independently of these two possibilities, in our model, we assume that anatomically and/or functionally distinct inputs from the hippocampus or the mPFC modulate the activity of BA fear and extinction neurons in a context-specific manner. That is, sub-populations of BA neurons are innervated by hippocampus/mPFC efferents that represent the current context. In addition, all BA neurons receive inputs from US/CS responsive LA neurons during conditioning and extinction. Those sub-populations of BA neurons that receive simultaneous LA and context-specific inputs become responsive during conditioning or extinction and, thus, emulate the “fear” and “extinction” neurons reported by Herry et al. [17]. Activation of BA neurons per se, however, is not sufficient to cause or prevent a behavioral response, but the selective activation of BA neurons conveys important information about the context-CS relation to the CEA. Although we do not model here the CEA, we stipulate that context-dependent BA activity provides an instructive signal to CEA neurons. In the CEA, it is likely that conditioning [18] and possibly extinction learning-induced changes act upon this signal in order to activate or suppress a fear response. If more experimental data, sufficient to constrain the possible parameter space, become available, then our present model of the BA could be extended to study the impact of context-dependent BA activity on learning-induced changes in the CEA as well.

We test the plausibility of context-dependent activation of BA neurons in two different approaches: first, in an abstract firing rate model; second, in a more realistic spiking neuron network (SNN) model of the BA. Based on the results of our model we provide plausible explanations for several experimental observations in fear and extinction learning and make specific, experimentally testable predictions.

## Models

### Experimental observations

**Figure 1:**
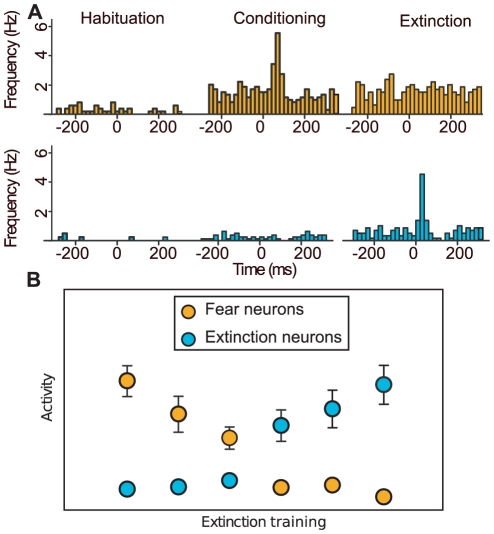
Fear and extinction neurons in rodents. (A) CS-evoked activity in the BA in pre-conditioning (left), post-conditioning (center) and post-extinction (right). After conditioning one subpopulation of neurons within the BA (fear-neurons, amber) increased their firing rates in response to the CS. This subpopulation did not show any CS evoked response after extinction. A different subpopulation (extinction neurons, cyan) did not respond to the CS during or after fear conditioning, but showed a CS evoked response after extinction training. (B) Population activities of fear and extinction neurons during extinction training for different blocks of CS presentations. In a different context, extinction training resulted in a progressive decrease in the response of fear neurons and increase in the response of extinction neurons. The switch of activity was correlated with a shift in behavior from high to low freezing. Figure adapted from [17].

The description of the evolution of the firing rates of BA neurons during fear conditioning and extinction reported by [17] provide certain simple, yet important, indications on the underlying dynamics in the BA network:

1. In naive animals, the ongoing activity of BA neurons does not predict the existence of different sub-populations of neurons.
2. As the animal learns to associate the CS with the US in 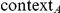 (the conditioning context), the activity of a sub-population of neurons in BA (fear neurons) increases, in correlation with the animal's freezing behavior (Fig. 1). The most parsimonious explanation suggests that the strength of a fraction of afferents carrying information on the CS (CS inputs from hereon) has been increased. Alternatively, changes in single neuron properties, e.g. excitability, or alterations in network activity states, e.g. reduced global inhibition, could also account for this observation.
3. During extinction training in a different context (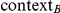), CS-induced activity of fear neurons progressively diminishes while the activity of a new sub-population of neurons (extinction neurons) increases. This suggests that during extinction, the strength of a new subgroup of CS inputs is strengthened, leading to the increase in response of the extinction neurons. Furthermore, the second and third observations highlight the importance of the context in the selective increase of the CS inputs. They also suggest that the strength of the contextual inputs to BA neurons may increase as well.
4. The sudden and selective increase of activity of fear neurons when the animal is put back in 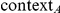 after extinction learning (i.e. renewal), reveals that extinction cannot be merely unlearning. Thus, the most simple explanation for the response reduction of fear neurons in 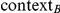 is local inhibition generated by the increased response of extinction neurons. It cannot be excluded, though, that partial unlearning - i.e. depotentiation of a certain fraction of previously strengthened synapses - may occur in parallel.

To test the feasibility of the above observations and their inferences in explaining the emergence of fear and extinction neurons in BA, we first studied the dynamics of a mean-field (or firing rate) model of the BA. Subsequently, we constructed a spiking neuron network (SNN) model to examine our hypotheses and their implications under more realistic conditions.

### Mean-field model of the BA

**Figure 2:**
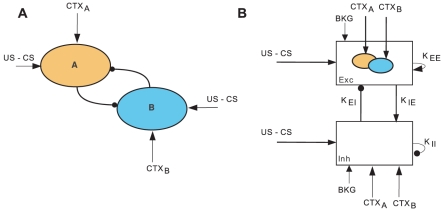
Schematic network model diagrams of the BA. (A) Firing rate model. Two neuron populations A and B are mutually coupled with negative weights. Both populations receive US-CS and context-specific (CTX) inputs. These external inputs can exhibit LTP. (B) Spiking neural network model. The network consists of 3400 excitatory and 600 inhibitory LIF neurons. The neurons are interconnected in a recurrent fashion. US-CS input is provided to all neurons. CTX input is fed only to two subpopulations of excitatory neurons. The external inputs (CS, US and CTX) are modeled as rate-modulated Poisson spike trains.

The mean-field model of BA consisted of two neuron populations, A and B, described by Wilson-Cowan type rate dynamics [19] (Fig. 2A). Both populations were *identical* in their properties (Eqs. 1–2) and received both CS input and non-specific background input. There is ample experimental evidence that in different contexts, different sets of hippocampal neurons (e.g. in CA1) are active [20]–[22]. Thus, to mimic context-specific inputs - either directly from hippocampus or indirectly via the mPFC or other brain structures such as entorhinal cortex - we provided population A with additional input 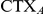 reflecting 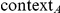, and likewise, population B with additional input 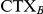 reflecting 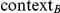. Populations A and B were mutually interconnected with inhibitory synapses. The system of differential equations describing the activity of the populations A and B is as follows:



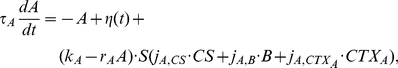
(1)




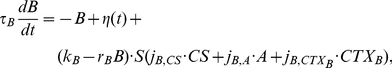
(2)where 
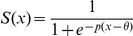
.

The evolution of the connection strengths is given by 
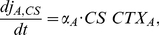
(3)




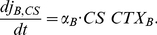
(4)


Here, 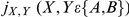 represents the connection strength from population (or external input) *Y* to population *X*, 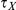 is the time constant governing the dynamics of population *X*, *k_X_* is the maximum firing rate of population *X*, and *r_X_* captures the refractoriness of neurons in *X*. The transfer function S is a sigmoid function, integrating all inputs to population *X* in a non-linear fashion and producing a bounded output rate. The parameters p and *θ* of the sigmoid function determine the steepness and the position of its maximum slope, respectively. The term *η*(*t*), with zero mean, reflects the stochastic input to the two populations, mimicking the background activity in the BA.

Equations 3 and 4 describe the dynamics of the connection strengths of the CS afferents onto populations A and B respectively. These weights were increased in an additive way whenever the respective CS and CTX inputs were present simultaneously and remained constant otherwise. The parameters *a_A_* and *a_B_* specify the learning rates (see also Eqs. 6–8).

We simulated fear conditioning and extinction by applying CS input to both populations in the form of short pulses of 50 ms duration each, based on the experimental design used in [17]. Contextual input was provided continuously. Note that we did not make any explicit distinction between the unconditioned stimulus (US) and conditioned stimulus (CS). Instead, we assumed that during conditioning, neurons in the LA initially responded to the US and eventually to the CS, while continuing to respond to the CS during extinction [23]. The output of these LA neurons was then fed downstream to the BA. In addition, US or CS inputs from the thalamus or the primary sensory cortex may directly target BA neurons [24]. In our model, we represented those inputs, independently of their origin, as CS-US in the conditioning context and CS in the extinction context.

### Spiking neuron network model of BA

For the description of the SNN we adopted the *good model description practice* proposed by [25], which provides guidelines for a standardized way of describing complex neural networks. We share the authors' belief that such model description facilitates reproducibility and direct comparisons between models. Within this framework, we organized the description in different subsections, complemented by additional information on the model parameters. This collected information is presented in an easily accessible, tabular form in the Supplementary Materials (Table S1).

Our choice to use leaky-integrate-and-fire (LIF) neurons was motivated by four major arguments: (i) multiple combinations of sub-cellular parameters can result in the same network state [26]; (ii) even simple neuron models such as LIF with minor modifications are sufficient to reproduce complex *in vivo* spike patterns [27]; (iii) realistically-sized large scale networks of LIF neurons can now be simulated with the currently available simulation technology [28]; this is hardly possible for similarly large networks built of detailed compartmental models and, finally, (iv) the extent to which sub-cellular properties of individual neurons influence the global network dynamics is presently not clear. Most importantly, however, here we are interested in understanding the key *network* level properties of the BA which play a critical role in the formation of fear and extinction memories. For this purpose, the LIF neurons, although they are reduced models of a biological cell, provide an adequate level of biophysical realism, sufficient to identify these key network properties.

### Network composition and connectivity

We modeled the BA as a random recurrent network, consisting of 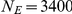 excitatory (EXC) and 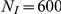 inhibitory (INH) neurons [24], [29]. A total number of 4000 neurons corresponds roughly to 10% of all neurons in the rat BA [30]. The schematic diagram of the network is shown in Fig. 2B. Each connection from a pre- to a post-synaptic neuron had an assigned probability, the value of which depended on the types of pre- and postsynaptic neurons involved (EXC and INH, respectively): 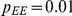, 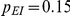, 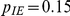, and 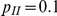. Thus, each EXC neuron received on average 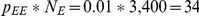 excitatory and 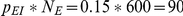 inhibitory connections. Likewise, each INH neuron received 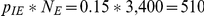 excitatory and 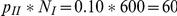 inhibitory connections. Neurons were allowed to form recurrent connections to themselves. For the simulations shown in the last figure, we systematically varied the connection probability of the recurrent inhibition from 0.1 to 1.0.

### Input, output, and free parameters

EXC and INH neurons received inputs encoding information on the CS. Similarly to the rate model, these inputs represented initial responses of LA neurons to combined CS and US presentations, later only to the CS. They might also reflect more peripheral, thalamic or cortical responses to CS-US. A fraction of BA EXC neurons (20%, randomly chosen) received inputs representing CS and 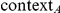. Similarly, another 20% of BA EXC neurons received inputs representing CS and 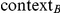. Thus, similar to the rate model, we assumed that BA EXC neurons receive contextual information directly from the HPC (or entorhinal cortex) and/or via the mPFC. Crucially, CS-US and contextual inputs converged onto the same neurons [8]. Furthermore, EXC and INH neurons received unspecific background inputs (BKG), representing activity originating in other areas, either within or outside the amygdaloid complex. The BKG inputs accounted for the baseline spiking activity of EXC and INH neurons at <1 Hz and 10–15 Hz, respectively [24].

The exact temporal and spatial patterns of the spiking inputs to the BA are not known. Here, we used independent Poisson spike generators with different firing rates to produce the specific inputs. Contextual and BKG inputs provided a tonic drive to BA neurons. By contrast, the CS input had a short duration of 50 ms, based on the experimental design used in [17]. All external inputs formed excitatory synapses onto their target neurons.

### Neurons, synapses, and plasticity

Neurons were modeled as leaky-integrate-and-fire (LIF) neurons. The subthreshold dynamics of each LIF neuron were governed by the following equation
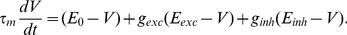
(5)


A spike was generated whenever the membrane potential crossed a predefined static threshold *θ* in upgoing direction. The potential was then reset to a value *E_k_* and clamped for *t_ref_* ms before the synaptic integration started again (Table S1F). Neurons made either excitatory or inhibitory connections onto their postsynaptic targets via conductance-based synapses [31]–[33].

The synapses of all connections were non-modifiable, except those providing CS and contextual input to EXC neurons. These latter, plastic synapses were modified according to the following phenomenological rule:



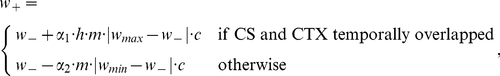
(6)




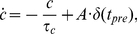
(7)




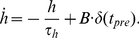
(8)


Note that three variables were used: the synaptic weight *w* and the auxiliary variables *c* and *h*. Each time a presynaptic neuron fired, the value of *c* increased by a fixed amount. Afterward, this value relaxed towards zero. Thus, variable *c* acted as a synaptic tag, encoding the recent activity in the synapse receiving CS input. Likewise, variable *h* encoded information about recent activity in neighboring synapses receiving contextual input.

**Figure 3:**
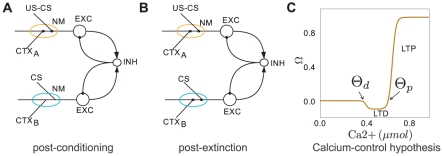
Synaptic plasticity. (A) and (B) Schematic connectivity diagrams of typical fear and extinction neurons in the BA network model (cf. Fig. 2B). We assume that US-CS and CTX synapses are plastic and spatially clustered on the same BA excitatory neuron (amber and cyan ellipses; NM: neuromodulator). (A) During fear conditioning, co-activation of CS, US and CTX afferents, strengthened the synapses (black dots) on fear neurons in the presence of a neuromodulator. (B) During fear extinction training, co-activation of CS and CTX afferents strengthened the synapses (black dots) on extinction neurons. Lack of CTX inputs resulted in a small depotentiation of CS synapses onto fear neurons (cf. Models). (C) The plasticity mechanism that drives the change in synaptic strength is essentially an implementation of the calcium-control hypothesis [Eqs. 6–8]. If CS and CTX inputs temporally coincide, the calcium influx in the neuron crosses threshold 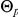 and LTP is induced in both CS and CTX synapses. By contrast, if only one of the CS and CTX inputs is active, the total calcium influx lies between 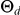 and 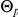 and the CS or CTX synapses exhibit LTD. If none of the inputs is active, the total calcium level stays below threshold 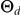 and the synapses remain unaltered.

At the offset of each CS presentation, the variables *c* and *h* were probed in the synapses of all EXC neurons and the strength of each synapse was modified accordingly. The synaptic strengths before and after the update are denoted by *w*_−_ and *w_+_*, respectively. If CS and contextual inputs at the same neuron coincided within a temporal window of ∼100 *ms*, then both synapses were strengthened [34]. By contrast, if only one of the inputs was present, both synapses were weakened (Eq. 6). This decrease of synaptic strength was based on studies reporting that synapses in LA, which had been strengthened during fear conditioning, depotentiated after extinction training [35], [36]. We assumed a similar mechanism to hold for the BA. This type of bidirectional plasticity rule implemented in our model is similar to the BCM rule [37], the “calcium-control hypothesis” [38]–[40] and the ABS rule [41], [42]. Common in all these rules is the specification that the level of postsynaptic *Ca* ^2+^ determines the direction of plasticity (for review see [43]). A large increase in *Ca* ^2+^ causes LTP, whereas a moderate increase results in LTD. Low levels of *Ca* ^2+^ do not cause any modification at all. We essentially incorporated this bidirectional induction of plasticity in our rule using fixed thresholds (Fig. 3C), rather than sliding ones, as is the case e.g. in the BCM rule. The parameters *a*_1_ and *a*_2_ denote the learning rates for potentiation and depotentiation of the synapses, respectively.

*Ca* ^2+^ influx depends on NMDA receptor activation and sufficient postsynaptic depolarization. The latter can be caused by coincident presynaptic input or by a backpropagating action potential (BAP). However, in our model, a BAP was not required. That is, we assumed that if the total presynaptic firing rates were high enough, they could cause sufficient depolarization to unblock NMDA receptors. This assumption is supported by experimental evidence showing that a BAP is neither necessary nor sufficient for synaptic plasticity [44], [45].

Note that this plasticity rule is also compatible with changes induced purely in the presynaptic terminal. In fact, experimental evidence suggests that presynaptic induction, completely independent of postsynaptic activity, occurs in the LA [46]. Thus, the plasticity rule implemented in our model incorporates both changes that are dependent on post-synaptic depolarization, but not postsynaptic spiking, and changes that are presynaptic and entirely independent of post-synaptic depolarization or spiking.

Because in our model the presynaptic spiking was caused by CS and contextual inputs, their total activity encoded in the variables *c* and *h*, respectively, determined the direction of plasticity. Thus, both *c* and *h* functioned as eligibility traces for synaptic modification [34], [47]. They could be interpreted as describing any relatively slow process associated with the effects of *Ca* ^2+^, e.g. autophosphorylation of CaMK-II [39], [48].

The terms 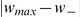 and 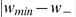 in the update rule were introduced to provide upper and lower bounds to the synaptic weights, such that they did not increase or decrease indefinitely. They also controlled the step-size with which synapses were modified: the closer a weight was to *w_max_* (*w_min_*) the smaller were its increments (decrements).

The parameter *m* represented the action of neuromodulators released during fear conditioning and extinction. It is known that many neuromodulators target the BA [5], possibly affecting synaptic plasticity in a complex way. Among the possible candidates are norepinephrine (NE) [49]–[52], dopamine (DA) [53], [54] and opioids [5]. Here, however, lacking more detailed experimental data, we cannot be more specific about which exact neuromodulators are involved and how they interact. Fortunately, this lack of knowledge does not pose a problem for the plasticity rule we propose, because it is general enough to accommodate any combination of neuromodulators that may turn out to be involved in BA fear processing.

The dynamics of the mean-field model were simulated in MATLAB. The SNN simulations were written in python (http://www.python.org), using the PyNN interface [55, http://neuralensemble.org/trac/PyNN] to the NEST simulation environment [56, http://www.nest-initiative.org].

## Results

### Firing-rate model of BA

**Figure 4:**
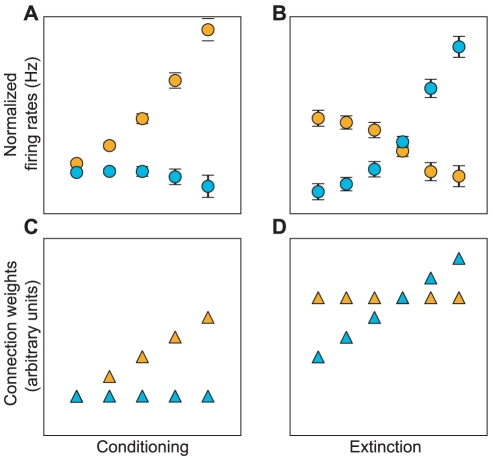
Dynamics of the firing rate model of BA. Five US-CS stimulations were used for conditioning and six CS stimulations for extinction. (A) During conditioning, LTP at US-CS and CTX afferents yielded increased activity of one of the sub-populations (amber dots). The increase in the activity of this sub-population resulted in a weak inhibition of the second sub-population (cyan dots). (B) During extinction, the same plasticity mechanism resulted in a gradual increase in the activity of the second subpopulation (cyan dots). This increase inhibited the first subpopulation, which also received less excitation in the extinction training. The normalized firing rates represent the average over 30 simulations. Note that the firing rates, although they have an upper bound, determined by the refractory term in Eqs. 1–2, remain far from saturation. (C) The evolution of synaptic weights 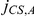 governing the increase in the firing rate of the fear neurons during fear conditioning (amber Δ). (D) Evolution of synaptic weights 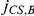 during extinction training (cyan Δ). A steady increase in the strength of synapses 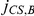 resulted in a steady increase in the firing rate of extinction neurons. In the firing rate model no depotentiation of synapses was implemented.

Fig. 4 shows the response of the mean-field model, i.e. the firing rate model, of BA during fear conditioning and extinction. To simulate fear conditioning in 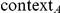, we stimulated the population A five times with CS, US and 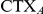 inputs (Eqs. 1,2). This resulted in a progressive strengthening of CS synapses onto population A (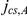) (Fig. 4C), accompanied by a corresponding increase in the response of population A (Fig. 4A). To simulate fear extinction training in 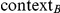, we stimulated population A with CS input and population B with CS and 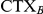 input six times to mimic a different context. Now, in 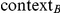, the synaptic strength of the CS input synapses (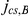) onto population B progressively got stronger, whereas 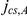 remained unchanged (Fig. 4D). The slow increase in the response of population B resulted in a small decrease in the response of population A, due to the recurrent inhibition. When the strength of 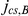 became larger than 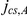 (Fig. 4D), the activity of population B dominated and, hence, the response of population A was suppressed (Fig. 4B).

The differential activation of two neuronal sub-populations in two different contexts can be interpreted as fear (population A) and extinction (population B) neurons as observed in [17]. This is purely a functional characterization of the two sub-populations, which are identical otherwise. That is, we used exactly the same parameters for both sub-populations and the differential activation results solely from differences in contextual inputs they receive. Thus, the two populations were not different in terms of their intrinsic properties. Of course, cases where the two subpopulations do have different properties can be easily accommodated in the model resulting in an enhancement of the differential activation. To be consistent with [17], we used the terms fear and extinction neurons to refer to those subpopulations that are active in 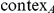 and 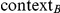 respectively.

Note that we did not include any component that imitates behavioral output, i.e. freezing. Instead, we assume, in agreement with experimental findings [17], that high activity of fear neurons directly corresponds to a high level of freezing whereas high activity of extinction neurons and low activity of fear neurons corresponds to low levels of freezing.

### Spiking neuron network model of BA

Although a simple firing rate model was able to account for the dynamic emergence of fear and extinction neurons, such mean-field models have only limited explanatory and predictive power. For instance, they assume uncorrelated activity in the underlying neuronal populations and, thus, cannot be used to predict any correlations in firing rate or spike timing that may emerge in the network. In addition, these models cannot be used to predict the spike patterns of individual neurons. Thus, to understand the dynamics of the BA network beyond average firing rates only, we simulated a biologically realistic large-scale network composed of spiking neurons. Again, fear conditioning and extinction were simulated by applying five CS-US presentations in 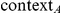 and six CS presentations in 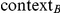 respectively. In the two different environments tonic contextual input was provided to EXC neurons (cf. Models).

**Figure 5:**
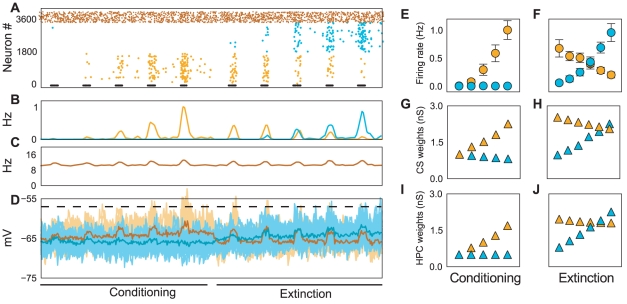
Dynamics of the spiking network model of BA. Five US-CS stimulations were used for conditioning and six CS stimulations for extinction. (A) Raster plot of the spiking activity in the SNN. Two different subpopulations of excitatory neurons (EXC) within the BA emerged during conditioning and extinction simulation, respectively. Neurons 1–1,700 correspond to the fear neurons (amber) and neurons 1,701–3,400 to the extinction neurons (cyan). Dark amber dots show the activity of inhibitory neurons. Short horizontal lines (black) at the bottom of the raster plot represent US-CS presentations (50ms) in fear conditioning and only CS input during extinction training. Observe that activity increased in the fear neurons during conditioning and was then suppressed during extinction training by a steady increase in the activity of the extinction neurons. Note that in contrast to the firing rate model, inhibitory neurons were explicitly simulated and played a critical role in the mutual competition between fear and extinction neuron subpopulations. The decline of fear neurons activity reflects the combined effects of active inhibition and unlearning (LTD at CS and CTX afferents). The increase of the baseline rates provides an explanation for contextual conditioning. Only 5 out of 6 CS stimulations shown for extinction training. (B) Firing rates of fear (amber) and extinction (cyan) neurons. (C) Firing rate of inhibitory neurons. Similar to the rate model, the rates of excitatory and inhibitory neurons remained low and far below the saturation level, determined by the refractoriness of the neurons. (D) Average free membrane potentials (spiking prohibited) of 100 randomly recorded fear (amber) and extinction (cyan) neurons. Twenty traces for individual neurons from fear (light amber) and extinction neurons (light cyan). The dashed black line shows the average spiking threshold (−57 mV) of the excitatory neurons. (E,F) Evolution of the firing rates of fear and extinction neurons during fear conditioning (E) and extinction (F). Firing rates were averaged over 30 simulations. (G,H) Evolution of synaptic weights of CS afferents onto EXC neurons during fear (G) and extinction (H) training, for the simulation shown in (A). (I,J) Same as in (G) and (H) but for the weights of CTX afferents to EXC neurons.

The results of the simulation are presented in Fig. 5. Initially, all EXC neurons spiked at very low firing rates. Presentations of the CS-US led to a steady increase in the firing rates of one sub-population (fear neurons) within the EXC population, which peaked at the end of conditioning (Figs. 5A, E amber dots). The increase in activity of fear neurons was a direct consequence of the potentiation of CS and contextual inputs onto fear neurons (Figs. 5 G, I; amber triangles). In 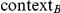, the fear neurons still responded with high firing rates upon the first CS presentation, even though they did not receive contextual inputs (Figs. 5A, F). With further CS presentations, however, 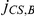 synapses became potentiated (Eq. 6, Figs. 5H, J; cyan dots), causing a steady increase in the firing rate of the second sub-population of neurons (extinction neurons) (Figs. 5A, F; cyan dots). The increased recurrent inhibition in the network then caused a decrease in the activity of the fear neurons (Figs. 5A–C, F). At the end of extinction, the population rate of the extinction neurons peaked, whereas the firing rate of the fear neurons had returned to the initial, pre-conditioning values. The reduction of fear neurons activity was further facilitated by small depotentiation of CS and contextual input synapses onto the fear neurons (Eq. 6, Figs. 4H, J; amber triangles). Note that depotentiation of CS synapses onto extinction neurons also occurred during conditioning (Fig. 5G) as described by the learning rule. By contrast, CTX synapses were not decreased during conditioning, because their initial values were close to the lower bound (*w*_−_) (Fig. 5I). During conditioning and extinction the baseline firing rates increased as well (Fig. 5A). This increase was induced by the strengthening of the contextual inputs (Figs. 5H, I), providing an explanation for contextual conditioning. However, because only a small percentage of neurons exhibited this increase in firing rates, this could make it difficult to measure it experimentally. This fact reveals a key advantage of network models which allow for simultaneously sampling a large number of neurons. Based on this, predictions can be inferred which otherwise would not have been possible.

Note that, again, the assignment of BA EXC neurons in fear and extinction sub-populations is purely a functional one. That is, neurons were characterized post-experiment as fear or extinction cells depending on whether they responded to the CS after conditioning or after extinction training respectively. In particular, they were not predetermined in terms of their intrinsic properties and the two sub-populations resulted solely from the differences in the contextual inputs they received.

**Figure 6 pcbi-1001104-g006:**
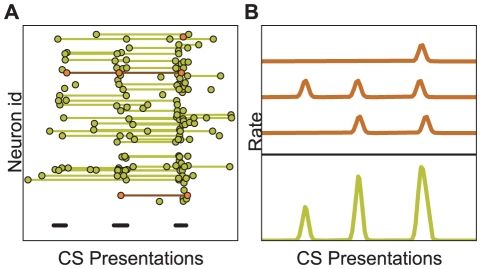
Single-neuron activity during fear and extinction. (A) Spikes (dots) of excitatory neurons for three consecutive CS presentations (short black lines). Spikes from the same neurons are connected by thin lines. Different neurons started to respond to the CS with 1–2 spikes at different points in time. Thus, the state of individual neurons did not change gradually, but quite abruptly from non-responding to responding. (B) The firing rates of three neurons from (A) (amber) that started responding to the CS at three different times. The average response of these three neurons yielded a gradual increase of activity (green), which does not reflect the abrupt response changes at the single-neuron level.

Also, it is important to emphasize that whereas the population rates of fear and extinction neurons increased gradually during conditioning and extinction training respectively, this was not the case for individual neurons. Instead, they changed their state quite abruptly from non-responding to responding (Fig. 6A). The further the training advanced, the more neurons started to respond. Hence, the gradual increase in population rates (Figs. 5E, F) reflects the growing recruitment of responding neurons, rather than a gradual increase of single neuron activity itself (Fig. 6). The responsive neurons fired maximally two spikes per CS presentation. The baseline firing rates for the inhibitory population were normally distributed with a mean of 10 Hz, whereas the CS-evoked rates shifted their distribution towards a mean of 20 Hz. This is consistent with the neuronal firing patterns *in vivo* reported by [17].

### Persistent neurons

Although we performed our main simulations using separated contextual inputs to distinct neuronal subpopulations within the BA (cf. Models), this is not a necessary requirement of the model. In fact, performing simulations with varying amounts of contextual input overlap showed that fear and extinction neurons still existed as distinct populations, even when contextual inputs had an overlap of around 50% (Fig. S1). In addition, the simulations revealed the existence of a third sub-populations of neurons. These were the neurons receiving inputs in both contexts and, thus, were active during both fear conditioning and fear extinction (so called persistent neurons). Note that, similar to the case of fear and extinction neurons, the characterization of cells as “persistent” is functional and denotes the fact that these neurons were responding to the CS during both conditioning and extinction. Moreover, these neurons had much stronger CS and CTX synapses, which resulted in higher firing rates. This observation of the model is consistent with the experimental data [17], suggesting that conditioning and extinction are not affected by overlapping inputs, unless the overlap is high (>50%).

### Renewal and extinction over-training

Following extinction training in 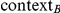, presentations of the CS in the original fear conditioning context (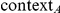) resulted in context-dependent renewal (ABA renewal) of conditioned fear responses [2]. This renewal phenomenon points at two important aspects of possible neural mechanisms underlying fear extinction: (i) extinction is mainly new learning and only partly unlearning of previously acquired fear memories ([57]; see also Discussion), (ii) extinction learning is context-dependent.

**Figure 7:**
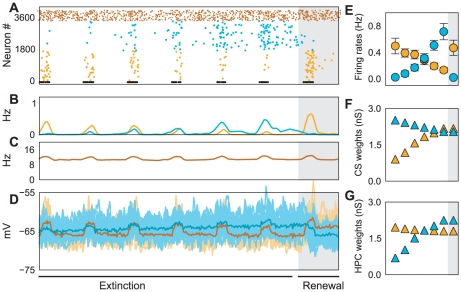
ABA fear renewal. (A) Spiking activity of fear (amber), extinction (cyan) and inhibitory (dark amber) neurons during extinction and renewal (gray shaded region). (B) Average firing rate of fear (amber) and extinction (cyan) neurons. (C) Average firing rate of inhibitory neurons. (D) Free membrane potential of fear and extinction neurons (cf. Caption Fig. 5 for more details). (E) Average firing rates of fear and extinction neurons during extinction and renewal (gray shaded region). (F) Evolution of synaptic strengths of CS afferents onto fear (amber) and the extinction (cyan) neurons. (G) Evolution of synaptic strengths of 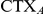 afferents onto fear (amber) and 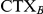 afferents onto the extinction (cyan) neurons. Switching context after extinction led to an instantaneous switch of activities between fear and extinction neurons (shaded gray regions in panels (A,B,E). In the initial conditioning 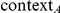 fear neurons dominated (due to 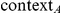 specific additional excitatory drive) and suppressed extinction neurons. Note that there was no change of weights during renewal (gray shaded area in panels F,G) revealing that the switch of activity was purely a network effect.

We simulated ABA renewal by changing context at the end of extinction (Fig. 7). This resulted in a sudden switch of activity between fear and extinction neuronal subpopulations. That is, although the activity of extinction neurons was high after extinction learning, the contextual switch caused the activation of fear neurons and a significant drop in the extinction neurons activity. These results are in complete accordance with the experimental findings reported by [17].

It is important to note that this rapid activity switch is purely a network phenomenon and not an effect of synaptic plasticity, as the change is much too fast for the plasticity mechanisms to act. We illustrate this point by depicting the average membrane potentials of 100 randomly selected fear and extinction neurons (Figs. 5D, 7D; amber and cyan traces respectively). It is evident that in either context there was a clear difference between the membrane potentials of the two cell populations, stemming from the fact that one of the populations continuously received a higher excitatory drive due to the additional contextual input. Switching contexts led to a corresponding instantaneous switch in the assignment of the contextual input and, hence, in opposite shifts in the average membrane potentials of the two sub-populations, which was immediately reflected in corresponding shifts in the firing rates.

We also modeled the case where the renewal context was different from both the conditioning and the extinction context (ABC renewal). The results of the simulations revealed that if after extinction training the CS was presented in a third, different 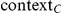, fear neurons became rapidly active again and suppressed extinction neurons (Fig. S2 middle). However, our model also indicated that the absolute response of fear neurons - and thus the magnitude of the fear response- would be weaker than in the ABA case. The reason is that in 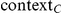 CTX synapses had not been strengthened during the conditioning procedure. This provides an account for the experimentally observed ABC renewal [58], [59] explaining why ABC renewal may occur in the first place and also why the effect may be weaker compared to ABA renewal.

Moreover, our simulations also suggested that massive extinction (extinction over-training) in 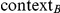 can abolish ABC renewal, because depotentiation of CS and 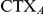 afferents onto BA neurons yield less excitatory input to these neurons. Extinction over-training can also impair ABA renewal, although to a lesser extent (Fig. S2 right). The reason that ABA renewal is more robust and ABC renewal more vulnerable to massive extinction stems from the fact that in the latter case not only CS synapses onto fear neurons are weakened, but also potentiated CTX synapses are entirely missing. These findings are in agreement with and provide a possible explanation for the experimentally observed effects of massive extinction [60].

### Extinction of contextual conditioning

Although we did not focus on extinction of contextual fear, it is important to note that our model also accounts for this specific conditioning phenomenon. Indeed, the plasticity rule dictates that in the absence of the CS synaptic weights will decay. That is, CTX synapses, which had been strengthened during conditioning in 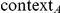 and encode contextual fear, will depotentiate in the same context if the CS is not present. This will yield decreased fear neuron activity and, thus, extinction of contextual fear. Note that within the framework of our model, this form of extinction is truly unlearning and not masking of contextual fear memories.

### High connectivity introduces gamma oscillations

**Figure 8:**
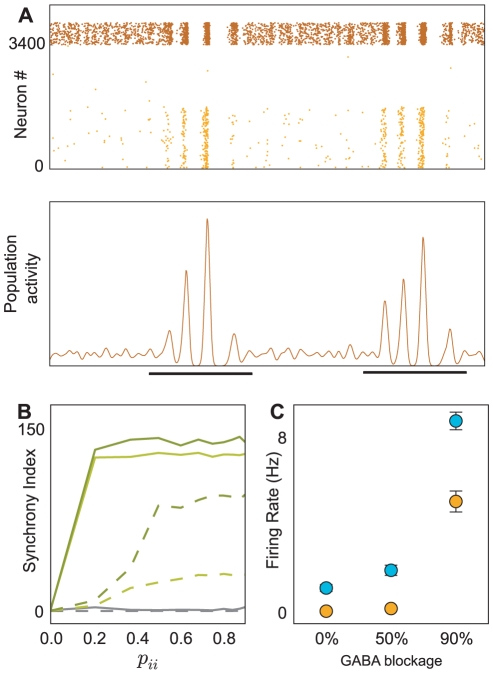
Synchrony and oscillations in the BA network model. (A) Spiking activity of a densely connected spiking neural network. A high connection probability from EXC to INH neurons as well as among INH neurons resulted in a population wide synchronization and gamma range oscillations, which were more salient during CS stimulation (black lines). (B) The effects of synaptic delays on the synchrony within the inhibitory population shown for synaptic strengths of 1 nS, 2 nS and 3 nS in gray, light and dark green, respectively. When synaptic delays were drawn from a uniform distribution [1–2 ms], increasing connectivity beyond 0.2 enhanced the oscillations and synchrony to their maximum (solid lines). Only for weaker synapses (1 nS) synchrony did not increase with connection probabilities, because the network was mainly input driven. Synchrony was significantly reduced for all synaptic strengths when synaptic delays were drawn from a uniform distribution in a lower delay range [0.2–1 ms] (dashed lines). However, note that for high connectivity (>0.4) and strong synaptic couplings, synchrony was always present. (C) Inactivation of INH neurons during extinction led to increased firing rates in both fear and extinction neurons (x-axis, percentage of inactive INH neurons). In 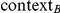, extinction neurons received additional contextual input and, thus, their activity was much higher (cyan) than that of fear neurons (amber). Independently of the behavior of the animal, the results in panel C suggest a simple experiment (i.e. blocking GABAergic synapses) to test the validity of the mechanism we propose here (see text, blockage of inhibition).

The experimentally reported connection probabilities from excitatory to inhibitory neurons as well as among inhibitory neurons in the BA are around 0.5 [61]. This is a much higher value than the ones we used in our initial simulations (Figs. 5–7, Table S1E). To test the effects of such higher connectivity, we performed additional simulations adopting the experimentally reported values for the connection probabilities. The qualitative behavior of the model did not change (data not shown). However, a new aspect in the network dynamics emerged. High frequency oscillations - typically in the gamma range (30–80Hz) - occurred throughout the simulation in both excitatory and inhibitory populations. These oscillations were present already in the ongoing activity patterns and CS-US presentation enhanced them even further (Fig. 8A). They resulted from the high shared connectivity and, hence, large amount of shared inputs that caused correlated spiking in the neurons. The oscillation frequency was determined by synaptic time constants and delays in the network.

Gamma oscillations in networks of excitatory and inhibitory neurons have been reported in many experiments [62]–[67] and discussed in multiple theoretical studies [68]–[75]. Moreover, several studies have reported gamma oscillations in the amygdala under certain conditions, e.g. in anesthetized animals [76], in slow wave-sleep [77], in the presence of reward predicting stimuli [78] and in paradigms involving consolidation of emotional memories [79]. Therefore, there is at least partial experimental and theoretical support for the gamma range oscillations observed here in high connectivity BA network simulations.

Yet, in networks with high mutual connectivity between excitatory and inhibitory neurons and within inhibitory neuron populations such as in the BA, oscillations should be a prevailing feature and should, therefore, be readily identifiable *in vivo* recordings under all conditions and not only in the special cases mentioned above. It is, thus, possible that certain mechanisms operate in the BA that could dampen gamma oscillations (but see Discussion). We, therefore, used our network model to investigate this issue in further simulations by exploring the parameter space of the network properties that could quench oscillations. Two mechanisms proved to be effective in reducing the power of gamma oscillations. The first one was the introduction of heterogeneity in the inhibitory population [80], [81]. This approach was motivated by experimental data showing that interneurons in the BA exhibit a large diversity in terms of their morphological and electrophysiological properties [24], similar to interneurons in the cortex [82] and hippocampus [83]. In the latter case, the diversity was expressed in a wide range of values for synaptic rise times, reversal potentials, response latencies etc. In a preliminary study [84], we introduced heterogeneity in one of the neuronal properties in our model, the spiking threshold, by drawing values from a bimodal distribution with peaks at −35 mV and −28 mV. This corresponds to the experimentally measured threshold values of two subclasses of parvalbumin-expressing interneurons in the BA: the fast-spiking (FS) and the delayed-firing (DF) interneurons [24], [61]. In such heterogeneous networks, oscillations were indeed reduced, but not totally eliminated [84].

A second, more effective way to reduce the network oscillations was to decrease the synaptic delays between inhibitory neurons (Fig. 8B). First, we studied the oscillations dynamics for different connection probabilities in a network of homogeneous neurons, with synaptic delays drawn from a uniform distribution (1–2 ms). In such networks, increasing connectivity (>0.2) enhanced the oscillations and synchrony to their maximum (Fig. 8B, green solid lines). Only for very weak synapses (1 nS), that is, when the network was mainly driven by external inputs, increasing the connectivity did not add to the oscillations (Fig. 8B, gray solid line). Increasing the width of the synaptic delay distribution did not reduce the synchrony and oscillations in high-connectivity networks (data not shown, Vlachos et al. in prep.). However, choosing short delays from a narrow uniform distribution (0.2–1 ms) considerably reduced the oscillations, up to connection probabilities of 0.4 (Fig. 8B, green and gray dashed lines). Thus, in a recurrent network, smaller delays have a powerful effect in reducing synchrony and oscillations. This finding is in agreement with a previous numerical study [69] and also with more recent analytical approaches [74], [75], [85]. At first sight, synaptic delays less than 1 ms might appear unrealistically small. However, delays as short as 0.5 ms have been reported among inhibitory neurons in the hippocampus [66]. Moreover, the delays between inhibitory neurons in the BA have been reported to be around 0.7 ms [61], or even smaller (Lüthi, unpublished data). Therefore these short delays, might indeed account for the lack of gamma band oscillations observed under baseline conditions in experimental recordings.

### Blockage of inhibition

Because inhibition plays a critical role in our model, we tested the effects of partial inactivation of inhibitory neurons. For this, we performed two additional sets of simulations, in which, during acquisition of extinction, we deactivated 50% and 90% of INH neurons, respectively. The results are shown in Fig. 8C. As expected, with reduced inhibition the activity of both fear and extinction neurons increased. The increase of activity of the latter population was more pronounced, due to the fact that it received additional excitatory drive from contextual inputs in 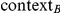. This suggests that blockage of inhibitory activity should lead to enhanced, context-specific extinction. This is consistent with the finding that GABA blockage enhances extinction of contextual freezing [86]. However, there is a potential caveat here. Activity of both fear and extinction neurons is increased upon blockage of inhibition and it is not clear how downstream structures, specifically CEA neurons, would respond to this. If the relative difference between fear and extinction neuron activity matters, then extinction should be facilitated by impaired inhibition. If, by contrast, the ratio between fear and extinction neuron activity is more relevant, then extinction might be impaired. Note that these two possibilities apply to both blocking of inhibition during acquisition of extinction training and blocking of inhibition during expression of fear extinction.

### Removal of contextual input

Because contextual input is one of the key aspects in our model we tested how removal of these inputs would affect the behavior of the network. The simulations yielded two different results depending on the exact time point of removal of contextual inputs. When contextual inputs were removed after fear conditioning, fear neurons remained active during extinction training and no extinction neurons emerged (Fig. S3; left). This result is a direct consequence of our synaptic learning rule, because strengthening of synapses requires temporal overlap of CS and CTX inputs. Note that although fear neurons remained active, their firing rates were reduced, because they now lacked contextual input. Thus, our model suggests that lesions of hippocampal or prefrontal areas after fear conditioning may result in impaired extinction. This conclusion is supported by experimental evidence [87].

By contrast, when contextual inputs were removed after fear extinction, activity of neither fear nor extinction neurons was sufficiently strong to suppress the other neuron group (Fig. S3; right). That is, because the decisive contextual input was lacking and, thus, both groups were simultaneously active, although to a lesser degree than in case either group was active alone. The behavioral consequences of these results are beyond the scope of our model, because here we did not model any downstream structures such as the central amygdala that presumably further process output from fear and extinction neurons. Thus, at present, we can only speculate that lesions of hippocampal or prefrontal areas after extinction training may result in impaired renewal, because fear neuron activity will be both decreased and also counteracted by simultaneous extinction neuron activity. In fact, experimental evidence supports this conclusion [88]. However, it is important to point out a subtle difference between our model and certain lesion experiments. In our model, removing CTX input means that the BA network does not receive any contextual input *at all*. By contrast, in some experiments in which the hippocampus had been lesioned or inactivated, contextual information may still have been accessible, because the context in which the CS was presented was still decisive for the behavioral outcome [89], [90].

### Predictions

Our model enables us to make a number of specific predictions that can be tested experimentally:

1. We predict that there is convergence of CS and CTX inputs onto the same BA EXC neurons. This prediction can be tested in multiple ways. One way is to look for anatomical connectivity. This can be achieved by employing pathway tracing studies. Note, however, that this approach can reveal stimulus convergence only on non-specific BA neurons, because fear and extinction neurons are only behaviorally determined in vivo. Alternatively, one could use optogenetic tools to activate/inactivate the LA, HPC or PFC while simultaneously performing intracellular recordings of BA neurons. If there is stimulus convergence, then activation (inactivation) of the connected structures would result in an increase (decrease) of the mean and/or variance of the membrane potential. A second way would be to test for functional connectivity, e.g. by using an imaging technique akin to the one used in the LA [91]. Again, these approaches can only reveal convergence on non-specific BA neurons. However, using electrophysiological techniques, the covariance of the spike rates between LA, HPC/PFC and *identified* BA fear and extinctions neurons could be measured while, again, selectively activating/inactivating LA or HPC/PFC. Functional convergence of CS and CTX inputs to BA fear and extinction neurons should be reflected in associated changes of the covariances.
2. Extinction over-training has a dual effect: (i) CS and 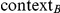 afferents to extinction neurons will become very strong, thereby enhancing the suppression of fear neurons. (ii) At the same time, depotentiation of CS and 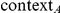 afferents onto fear neurons will substantially decrease the excitatory input to these neurons. This should be visible during renewal, where presentation of the CS in 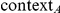 or 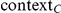 should lead to only a weak fear response (Fig. S2; right). Existing experimental results seem to support this potential effect of massive extinction [60]. Here we predict, in addition to these behavioral findings, the state of ‘hidden’ or not directly observable variables (large synaptic weights) and explain how they may affect direct observables (enhanced extinction).
3. Conditioning and extinction training increase the excitatory and inhibitory inputs to principal neurons in the BA. This results in stronger fluctuations of their membrane potentials. Thus, the variance of the recorded membrane potential fluctuations of BA neurons in trained animals should be higher than in naive animals.
4. Blockage of inhibition in 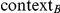 results in elevated activity in both fear and extinction neurons, with extinction neurons spiking at a higher rate than fear neurons. It is not clear how this may impact on downstream structures such as the CEA. Irrespective of the behavioral outcome, a specific experiment addressing the effects of GABA blockage on fear and extinction neurons activity would provide more insights into this issue.
5. The strength of fear (extinction) memories is directly proportional to the strength of contextual inputs to the BA during conditioning (extinction) training, respectively. If salience of environmental features translates to higher firing rates or increased number of contextual inputs to the BA, then renewal (extinction) will be enhanced. Along the same lines, the bigger the differences between conditioning and extinction context, the more extinction should be facilitated, because the overlap of contextual inputs to the BA between the two contexts will be minimized.

## Discussion

Here we presented for the first time a large-scale network model of the BA addressing the question how contextual inputs may shape the activity of distinct sub-populations of BA neurons. Although we started from a very specific experimental data-set [17], we implemented a network model that has more far-reaching implications. That is, the results of the simulations together with the model architecture provide, non-trivial and experimentally testable new insights into potential neural mechanisms underlying cued and contextual fear conditioning and extinction, ABA and ABC renewal, and extinction over-training. In addition, a specific and important function of inhibition is sketched as a mechanism that could enable mutual competition between fear and extinction memories. These results allow us to provide a synthesis of several experimental findings and to propose a role for the BA as a nucleus that integrates information about the CS and the context. This brings it into the position to provide a context-dependent instruction to downstream structures, enabling the switching of states from low to high fear and vice versa.

Specifically, we propose that context modulates neuronal activity within the BA, resulting in the formation of associations between CS, US and context in this nucleus. During fear conditioning the 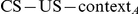 representation signals danger and causes a high fear state. During extinction, the newly formed 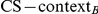 representation signals safety and suppresses the fear state. Back in the conditioning context, the initial representation dominates again (renewal). Thus, as far as neural mechanisms within the BA are concerned, conditioning and extinction could be understood as mutual competition between different representations of fear and safety. Partial unlearning or erasure may also occur, although to a limited degree.

Memories are assumed to be stored in a distributed manner in the brain [92]. Consistent with this view, fear-related memories may also be distributed among different nuclei within the amygdala and brain regions connected to it [10]. Our model suggests that context-related features of these distributed fear memories are represented in the BA. Thus, inactivation of the BA would impair context-related aspects of fear and extinction memories, whereas non-contextual features, represented in LA or CEA, would remain unaffected [17].

### Sources of contextual inputs

One core feature of our model is that contextual inputs are gated to the BA. In this framework, the precise origin of these inputs does not matter; as long as the BA neurons receive differential inputs in two different contexts, the model behavior remains unaltered. However, there are strong indications from anatomical [29], [93], [94] and physiological [93] studies that the HPC is a major source of contextual information to the BA. In addition, a previous report showed context-dependent modulation of neuronal activity in the LA [95]. By designing our model to have contextual input directly influencing the activity of excitatory neurons in the BA, we have essentially postulated a similar mechanism for this subnucleus. This assumption is further supported by the finding that fear neurons show orthodromic responses to HPC stimulation [17].

A second source of contextual input may be the mPFC. There is anatomical evidence that the mPFC projects to the BA [29]. Moreover, [17] reported that mPFC stimulation induces orthodromic responses in identified extinction neurons. Here, we suggest that part of the information conveyed by these projections might be contextual. This assumption is based on evidence reporting extinction-related induction of LTP on hippocampus-mPFC afferents [96]. In our model both fear and extinction neurons receive context-specific information either directly from hippocampus or indirectly via the mPFC. This may also explain the ambiguous results that the hippocampus may or may not interact with the mPFC during extinction learning [97].

The context-specific modulation of activity in the BA presented here provides a general framework that can explain experimental findings on the involvement of the hippocampus in the acquisition, encoding, and context-dependent retrieval of both conditioning [13], [98], [99] and extinction memories [3], [87]. Future refinements of the model, in combination with new experimental data are necessary for a better understanding of the detailed interactions between hippocampus, mPFC and amygdala.

### Gamma oscillations

We showed that high connectivity between excitatory and inhibitory and within inhibitory neuron populations results in robust oscillations in the gamma range, characterized by high activity correlation among neurons. The main cause of these oscillations was the high degree of shared inputs among neurons as a result of the dense connectivity. We suggested two different, biologically plausible ways to reduce these oscillations: by either introducing heterogeneity in neuron properties and/or by reducing synaptic delays to sub-millisecond time scales. Yet another way would be to have synapses exhibit a certain transmission failure rate [100], [101], resulting in activity dependent reduction of the effective connectivity. However, we do not wish to imply that gamma oscillations do not exist in the BA. In fact, as noted earlier, gamma oscillations have been reported in the amygdala under various conditions [76]–[79]. Here, we want to emphasize the point that in networks with high connectivity, gamma range oscillations are a salient feature of the network dynamics. Therefore, they should be visible even in the ongoing activity, unless suppressing mechanisms, such as those elaborated here, are in effect.

Several suggestions for a specific role of gamma oscillations have been made in the past. For instance, it has been proposed that in the cortex or the hippocampus oscillations might contribute to temporal encoding [102], sensory binding [103], attentional selection [104] and memory formation or retrieval [105], [106]. It is currently unclear whether these hypotheses also apply to the amygdala. Oscillations in lower frequency ranges (delta and theta) have also been reported. For example, increased theta oscillations - that synchronized with hippocampal theta activity - were shown to be related to conditioned freezing [107], [108], whereas delta oscillations have been implicated in gating aversive stimuli [109]. Gamma oscillations, on the other hand, have been suggested to facilitate interactions between the amygdala and connected structures [78], [110].

Here, because we modeled only the BA, we cannot give any informed predictions about how gamma oscillations may affect those various interactions. Moreover, in our current model, we have used plasticity only in the input connections and those are not affected by oscillatory activity in the recurrent network. However, before addressing the effects of gamma oscillation on the dynamics of the BA network, it is of key importance to resolve experimentally whether gamma oscillations are indeed present in BA activity and, if so, under which conditions.

### Conditioned inhibition

A well-known behavioral phenomenon is conditioned inhibition, referring to the ability of a second CS (CS−) to suppress the conditioned response, after it has been paired several times with the first CS (CS+) in the absence of a US [57], [111]. It is possible that the CS−, referred to as conditioned inhibitor, employs similar mechanisms to those described in our model to suppress the conditioned response. That is, neural subpopulations in the BA encoding the CS− might, similar to extinction neurons, use local inhibitory circuits to suppress fear neuron activity. Future work is needed to explore further this interesting line of reasoning.

### Conditioning and extinction in the same context

Our model accounts for experimental paradigms that use a different extinction context from the conditioning one, but not for those in which fear conditioning and extinction occur in the same context. For instance, if conditioning and extinction both occur in 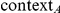, then only those neurons that receive inputs in this context will be active. Thus, downstream structures will not be able to differentiate between fear conditioning and extinction training solely from spiking activity in the BA. It is evident that performing conditioning and extinction in the same context *per se* increases ambiguity about the meaning of the context. Thus, it is likely that circuits within the BA alone are not sufficient to solve this computational problem. Both, a detailed description of neural activity during this type of extinction and a more detailed analysis of interactions between the BA and downstream structures are required to address this behavioral phenomenon.

### Relation to other models

Although a wealth of experimental studies exist on the amygdala and its role in fear conditioning and extinction, computational or theoretical approaches to study amygdala function are largely lacking. Most of the previous theoretical studies involve symbolic models [112], [113], mainly based on the Rescorla-Wagner rule [114]. These models have their merit in describing behavioral findings such as generalization, blocking etc. However, since these models treat the amygdala as a “black-box”, it is not within their scope to account for neuroanatomical or electrophysiological data, therefore providing little insight into the underlying neuronal mechanisms involved. Despite these apparent differences, it is still possible to draw some parallels to symbolic models. For instance, in our model, potentiation of synapses occurs only if CS and CTX inputs temporally overlap. This is similar to the SOP model, where US and CS have to coincide for strengthening of associations to take place [115], [116].

Connectionist or parallel-distributed (PDP) models of fear related processes go one step further than symbolic models by introducing networks composed of multiple, mutually connected computational units. One such model was successful in capturing certain features observed in fear conditioning studies [117]. Its main limitation, however, is the fact that it does not take into account the different substructures within the amygdala, nor do the computational units used in the model map to any biophysically realistic counterparts.

Fortunately, the computational power presently available allows us to improve these models and to overcome many of their limitations. The model presented here is to our knowledge the first large-scale spiking neuron network model that investigates the mechanisms of fear conditioning and extinction within the amygdala using biologically realistic neurons in adequate detail. The model closest to this is a compartmental model introduced by [118] to investigate the function of the LA in fear conditioning and extinction. However, [118] used a small network composed of only eight two-compartment neurons and focused on role of the kinetics of multiple ionic currents in fear conditioning and extinction. By contrast, we modeled the BA using a large network of 4000 LIF neurons, which enabled us to identify the network level interactions involved in the formation of fear and extinction memories.

### Conclusions

The present model provides a plausible explanation for the neural mechanisms underlying fear conditioning and extinction within the BA. We did not address the question of how the neural activity within the BA impacts on downstream structures, such as CEA or mPFC. We neither attempted to model the interactions between hippocampus and mPFC in conditioning and extinction, which would require additional experimental data to constrain the possible models. Given these restrictions, we provided a plausible mechanism of how contextual inputs may affect the activity of distinct neuronal subpopulations in the BA, enabling them to control downstream structures such as the CEA. We proposed that context-related aspects of fear and extinction memories are partially stored in the BA and that they provide a context-dependent instruction for the triggering or blocking of the fear-response. In addition, we showed how extinction training may mask previously acquired fear memories and, thus, provided an account for renewal. Finally, our model, next to yielding several interesting predictions discussed above, raises the important question of how downstream structures such as the CEA or mPFC discriminate the activity of the distinct neuronal subpopulations within the BA. Is this problem solved purely on an anatomical level, e.g. by differential projections of the BA subpopulations to specific target neurons? Or do specific features in the activity of the BA subpopulations, e.g. the statistical structure of pairwise or higher-order correlations, also play a role, providing downstream networks with a mechanism to distinguish between them? These questions need to be addressed in future work combining experimental and theoretical approaches.

## Supporting Information

Figure S1 Overlapping contextual inputs. (A) Venn diagram illustrating overlap of CTX inputs. (B) Activity of BA neurons at the end of extinction training. Varying the overlap of CTX inputs to BA neurons from 0–100% resulted in a third subgroup, which was active both during conditioning and extinction (persistent neurons). The higher the overlap, the more neurons in this group were active, as is reflected in the population rate. Note, that fear and extinction neurons still existed with the latter ones suppressing the former ones, even for an overlap of >50%.
(0.13 MB EPS)

Figure S2 ABC renewal and extinction over-training. After fear extinction in context B the activity of extinction neurons (cyan) was high, thereby suppressing the activity of fear neurons (amber) (left). Presenting the CS in the conditioning context-A resulted in a rapid switch of activity, with fear neurons suppressing extinction neurons activity (ABA renewal; see also Figure 7 and main text). When the CS was presented in a different context-C, then again fear neurons were active (ABC renewal). However, their activity was decreased compared to ABC renewal, because potentiated contextual input was now missing (middle). Our model suggests that extinction over-training can abolish both ABA and ABC renewal (right; see text).
(0.06 MB EPS)

Figure S3 Removal of CTX input. (left) Effects of removal of contextual input on fear and extinction neurons activity. When contextual inputs were removed after conditioning, then during extinction simulation no new group of neurons became active and, therefore, fear neurons remained active, as they were the only group of neurons for which CS inputs had been potentiated during conditioning. Thus, the model predicts that lesions of hippocampal or prefrontal areas after conditioning may result in impaired extinction. (right) When contextual inputs were removed after extinction, then both fear and extinction neurons were active and no group was able to suppress the other. However, the firing rates of both groups were decreased, because contextual input was lacking. The behavioral implication of these results may be impaired ABA as well as ABC renewal (see main text).
(0.12 MB EPS)

Table S1 Model parameters. Parameters used in the rate and SNN model.
(0.13 MB PDF)
