# Supplementary material for: Context-Dependent Encoding of Fear and Extinction Memories in a Large-Scale Network Model of the Basal Amygdala
Source: PLoS Comput Biol. 2011 Mar 17;7(3):e1001104. doi: 10.1371/journal.pcbi.1001104 (PMC3060104; doi:10.1371/journal.pcbi.1001104)
Supplement: Table S1 — Model parameters. Parameters used in the rate and SNN model. (0.13 MB PDF) [file pcbi.1001104.s004.pdf]

Table S1

Table S1A. Rate Model summary

Type	Wilson Cowan
Transfer function	Sigmoid logistic function $S(x) = \frac{1}{1+e^{-p(x-\theta)}}$, $p = 1.2$, $\theta = 2.8$
maximum rates	$k_A = k_B = 0.97$
refractoriness	$r_A = r_B = 10^{-3}$
learning rates	$a_A = a_B = 0.15$

Table S1B. SNN Model summary

Populations	two: excitatory neurons (EXC), inhibitory neurons (INH)
Topology	none
Connectivity	Random divergent connections prescribed by experimental findings
Neuron model	Leaky-integrate-and-fire, fixed threshold, absolute refractory period (2ms)
Channel models	-
Synapse models	Conductance-based difference of exponentials (AMPA, $GABA_A$)
Plasticity	CS and contextual projections onto EXC neurons
Input	all neurons: conditioned stimulus (CS), background input (BKG),<br>EXC receive in addition contextual (CTX) input
Measurements	Membrane potential, spike rates

Table S1C. Populations

Name	Elements	Size
EXC	LIF neuron	3400
INH	LIF neuron	600

Table S1D. Topology

none

Table S1E. Connectivity

Projection	Type	Connection probability
EXC to EXC	Divergent connections	0.01
EXC to INH	Divergent connections	0.15 (0.5)
INH to EXC	Divergent connections	0.15 (0.5)
INH to INH	Divergent connections	0.1 (0.1-0.9)
Weights		
EXC to EXC,INH	Static, drawn from normal distribution with $\mu = 1.25$ nS and $\sigma = 0.1$nS	-
INH to EXC,INH	Static, drawn from normal distribution with $\mu = 2.5$ nS and $\sigma = 0.1$nS	-
CS/CTX to EXC	Plastic, drawn from normal distribution with 1 nS and $\sigma = 0.1$nS	-
CS to INH	Static, drawn from normal distribution with 1 nS and $\sigma = 0.1$nS	-
Delays		
All connections	Fixed, drawn from normal distribution with $\mu = 2$ms and $\sigma = 0.1$ms	

Table S1F. Neuron Model

Name	LIF neuron
Type	Leaky integrate-and-fire, conductance based synapses
Subthreshold dynamics	$\tau_m \frac{dV}{dt} = (E_0 - V) + g_{ex}(E_{ex} - V) + g_{inh}(E_{inh} - V)$
Spiking	If V(t-) $<\theta \wedge$ V(t+) $>\theta$,<br>1. set t* = t<br>2. emit spike with time-stamp t^*<br>3. reset $V(t) = E_K$<br>4. clamp V(t) for 2 ms

Table S1G. Synapse Models

AMPA/GABA$_A$	$g(t) = g_{peak} \frac{e^{-t/\tau_1} - e^{-t/\tau_2}}{e^{-t_{peak}/\tau_1} - e^{-t_{peak}/\tau_2}}$

Table S1H. Plasticity

Description	Phenomenological rule of synaptic weight modification
Update rule	$w_+ = w_- + \alpha_1\, h\, m\, \lvert w_{max} - w_- \rvert\, c,$ if h>0<br>$w_+ = w_- - \alpha_2\, m\, \lvert w_{min} - w_- \rvert\, c,$ if h=0
Variables	$\dot{c} = -\frac{c}{\tau_c} + A\, \delta(t_{pre})$<br>$\dot{h} = -\frac{h}{\tau_h} + B\, \delta(t_{pre})$
Parameters	m: (non-specific) neuromodulator<br>m=1: neuromodulator present<br>m=0: neuromodulator absent<br>learning rates $a_1 = a_2 = 16 * 10^{-4}$<br>$(w_{max} - w_-)$: step-size increase

Table S1I. Input

Type	Description
CS to EXC/INH	One Poisson generator per neuron in EXC and INH, phasic drive: 50ms, spiking rate: 500 Hz
CTX to EXC/INH	One Poisson generator per neuron in EXC, tonic drive, spiking rate: 300 Hz
BKG to EXC	Current injection: $DC = 330$ pA , $AC = 85$ pA
BKG to INH	Current injection: $DC = 220$ pA , $AC = 110$ pA

Table S1J. Measurements

Membrane potential V and spike times for randomly selected neurons in EXC and INH
